# Epidemic Mitigation and Marginal Mortality Gains Using Self-Testing as a Diagnostic Intervention for Epidemic-Prone Diseases in Africa

**DOI:** 10.3390/diagnostics16132092

**Published:** 2026-07-03

**Authors:** Yasmin Dunkley, Elizabeth L. Corbett, Nicola Desmond, Pitchaya Indravudh, Nimalan Arinaminpathy

**Affiliations:** 1 Department of Clinical Research, Faculty of Infectious and Tropical Diseases, London School of Hygiene and Tropical Medicine, London WC1E 7HT, UK; liz.corbett@lshtm.ac.uk; 2 Botswana Sexual Reproductive Health Initiative, Botswana Harvard Health Partnership, Gaborone Private Bag BO 320, Botswana; 3 Department of Global Health and Development, Faculty of Public Health and Policy, London School of Hygiene and Tropical Medicine, London WC1H 9SH, UK; nicola.desmond@lshtm.ac.uk (N.D.); pitchaya.indravudh@lshtm.ac.uk (P.I.); 4 Faculty of Medicine, School of Public Health, Imperial College London, London SW7 2AZ, UK; nim.pathy@imperial.ac.uk; 5 Global Tuberculosis Programme, Department for HIV, Tuberculosis, Hepatitis and Sexually Transmitted Infections, 1211 Geneva, Switzerland

**Keywords:** self-testing, epidemic-prone diseases, diagnostic prioritization, outbreak preparedness, health-system constraints, modeling, Africa, diagnostic access

## Abstract

**Background/Objectives:** African Union (AU) guidance identifies decentralized diagnostics as central to epidemic preparedness. However, the epidemiological role of self-testing across epidemic-prone diseases remains underexplored. Drivers for the potential impact of self-testing were examined conceptually using a transmission model. **Methods:** A deterministic SEIR model compared standard-of-care testing with additional self-testing. Global sensitivity analysis using Latin Hypercube sampling and partial rank correlation coefficients (PRCCs) examined parameters influencing reductions in peak disease prevalence (mitigation). Dynamics were illustrated using AU pathogen archetypes (Ebola, Influenza A, Cholera, Coronavirus, and Mpox), estimating the number needed to self-test (NNST) to avert one death. **Results:** Epidemic mitigation was minimal (median 1.9%; IQR: 0.4–5.8%); this correlated with isolation adherence (PRCC *=* 0.784), self-testing intensity (PRCC *=* 0.617), lower R_0_ (basic reproductive number; PRCC *=* −0.607) and greater duration of infectiousness (PRCC *=* 0.370). Conditional scenario exploration indicated 34 self-tests per 10,000 people per day to achieve a 10% reduction in peak prevalence at R_0_ = 1.1, assuming self-test sensitivity 78.7%, specificity 99.3%. This exceeded the WHO Afro COVID-19 operational benchmark of 10 per 10,000 per week. High-mortality, moderate-transmission archetypes (e.g., Ebola) were most responsive to mortality reductions (median 1512 NNST/death averted) compared to Mpox (median 355,708 NNST/death averted). Adherence to post-test isolation exerted greater epidemiological impact than diagnostic accuracy. **Conclusions:** The epidemiological value of untargeted self-testing depends on pathogen characteristics and post-test behavioral adherence. Epidemic mitigation effects were limited under constrained health-system capacity. Future studies evaluating early decentralized self-testing deployment during Ebola-archetype outbreaks may identify operationally feasible deployment strategies to support mitigation and mortality reduction.

## 1. Introduction

African Union (AU) guidance identifies decentralized diagnostics as central to epidemic preparedness [1,2]. Self-testing as a diagnostic intervention is when an individual performs their own test, from sample collection to result interpretation at a time and place of their choosing [3], arguably constituting the most decentralized of diagnostic modalities. There is a wide literature demonstrating that self-testing can increase uptake and access in the context of HIV across Africa [4,5,6], alongside emerging evidence from Hepatitis C (HCV) on the continent [7,8]. A major shift in international self-testing policy and practice occurred during the COVID-19 pandemic [9,10,11,12,13]. The WHO interim and AU COVID-19 self-testing policy drew directly on evidence generated for HIV self-testing to recommend self-testing for COVID-19 for low- and middle-income countries [14,15]. However, despite the shared policy background, SARS-CoV-2 infection dynamics differ fundamentally from HIV and HCV. As an acute respiratory infection with a short infectious period and high self-cure rate, the value of testing is closely tied to the timely identification of infectious individuals to interrupt transmission [16].

In turn, despite mass-deployment and demonstrated clinical utility and epidemiological impact in high-income settings with COVID-19 self-testing [17], the evidence on COVID-19 self-testing in Africa is less clear. In high-income settings, epidemiological impact from repeated high-frequency self-testing at scale was against a background of high levels of existing diagnostic provision [18]. Across Africa, traditional models of diagnostic testing are characterized by a centrally controlled public health model with limited access and constrained capacity to achieve high testing intensity at population level [19]. During the COVID-19 pandemic, the World Health Organization Regional Office for Africa (the WHO Afro) published a universal benchmark of 10 tests per 10,000 people per week to disrupt transmission chains [20]. By mid-2021, only 16 countries had achieved this benchmark [21]. When COVID-19 self-testing policy was released by the African Union, it cited the potential benefit that self-testing could offer in bridging these system gaps, as a diagnostic access tool for preparedness [15].

More broadly, there is limited evidence and conceptual clarity on the potential epidemiological impact of self-testing for epidemic-potential pathogens on the continent. Self-testing has not been considered as an explicit component of decentralized diagnostics within preparedness frameworks, nor has research on self-testing and epidemic mitigation been prioritized in post-COVID-19 outbreaks such as the October-2024 Mpox outbreak [1,22,23,24]. Whether self-testing can contribute to epidemic mitigation is likely conditional, dependent on pathogen attributes, epidemiological context, achievable testing intensity, and post-test behavioral responses which, together, determine the rate at which infections can be identified and transmission interrupted. It remains unclear whether self-testing can achieve transmission impact under realistic conditions of coverage and adherence in resource-constrained settings.

This study applies a deterministic SEIR model to conceptually identify the drivers under which self-testing, in addition to the standard of care at low rates of background case-detection by the health system, could meaningfully mitigate an epidemic and avert deaths through reductions in transmission. This is not the only potential value of decentralized diagnostics; testing can support individual patient management and linkage to care, and it can foster timely identification of outbreaks and rapid response, as well as preventive benefit. However, this conceptual exploration focuses on epidemic mitigation as a first step in understanding the broader role of self-testing for epidemic-prone pathogens.

## 2. Materials and Methods

### 2.1. Model Structure and Specification

We developed a deterministic SEIR model to explore the impact of self-testing (ST) in addition to the capacity of the health system to detect cases [standard-of-care (SoC) testing]. Impact is defined as (i) epidemic mitigation (the relative reduction in the peak disease prevalence with the addition of self-testing compared to SoC), and (ii) the number needed to self-test (NNST) to avert one death as a measure of marginal efficiency. Parameters vary across pathogen, device, intervention, and behavior ranges, informed by the literature. The full model specification, list of differential equations and parameter definitions are provided in the Supplementary Information (Section S1). The model is deliberately simplified, aimed at providing conceptual insights that may apply across a range of infections. However, where empirical parameter decisions are required, we refer to policy priorities and data from the African region, given the urgency of strengthening preparedness on a continent facing the dual burden of increasing infectious disease outbreaks and constrained health systems [1].

### 2.2. Self-Testing and the Role of Isolation

The model captures how self-testing modifies the rate at which infectious individuals are detected and isolated.

Individuals progress from susceptible (S) → exposed (E) → infectious (I) → recovered (R) or death, governed by per capita transition rates ε (latent progression), γ (recovery) and μ (infection-specific mortality). A parallel ‘isolating pathway’ (*Š*, *Ẽ*, *Ĩ*, *Ř*) receives inflows from self-testing and SoC. Infectious individuals enter isolation through SoC at rate (r) and through self-testing at rate σ × sensitivity, where σ is the per capita rate of self-testing. The model also allows for false-positive test results from S, E and R, which enter isolation through self-testing at rate σ × (1 − specificity); SoC detection is assumed 100% specific. Isolated states return to the community at rate δ.

Adherence to post-test isolation influences transmission dynamics, as individuals may not fully curtail social contact during isolation and may also end isolation earlier than required. The parameter a captures adherence, ranging from 0 (no adherence to isolation) to 1 (full adherence). The force of infection was defined as $\lambda =(\beta I+(\left(1-a\right)\beta \tilde{I}))$ for non-isolated susceptibles and as $\tilde{\lambda }=(1-a)\left(\beta I+((1-a)\beta \tilde{I}\right))$ for isolated susceptibles. This construction captures both the reduced opportunities for transmission amongst infectious people that are isolated (through the term multiplying *β*) and the reduced risk of infection amongst susceptible individuals who are isolated (through the additional scaling of the force of infection by (1−a) when defining $\tilde{\lambda }$). Adherence also determined the proportion of the isolation period completed by individuals, with lower adherence resulting in an earlier exit from isolation (see Supporting Information).

### 2.3. Global Sensitivity Analysis

Simulation parameters are listed in Table 1. For any given parameter set, we simulated the epidemiological impact of self-testing as the reduction in peak disease prevalence, relative to a comparator of no self-testing. We used Latin Hypercube Sampling (LHS) to generate independent values for all parameters except for diagnostic accuracy parameters (sensitivity, specificity). These were sampled jointly from a bivariate posterior fit to SROC data to retain typical empirical correlations between sensitivity and specificity (Section S2). Specificity was capped above 0.90 to avoid rewarding devices with sub-optimal specificity (peak prevalence declines with increased false-positive isolation). We thus generated 20,000 parameter sets within plausible ranges defined from the literature, and hypothetical-yet-plausible pandemic-potential pathogen scenario modeling [25] (Table 1). We conducted a global sensitivity analysis (*N* = 20,000 simulations) using partial rank correlation coefficients (PRCCs) to identify generalizable drivers of epidemic mitigation, identifying parameters most correlated with reductions in peak disease prevalence.

Given that SoC testing (r) may modify intervention impact, we complemented the global PRCC with stratified PRCCs computed within deciles of r (Section S5).

We conducted a conditional scenario exploration at the lowest SoC testing and transmission scenario (R_0_ = 1.1), fixing all other parameters at the median values from the global sensitivity analysis to define operationally realistic self-test intensity thresholds for epidemic mitigation. This step motivated subsequent exploration of marginal-efficiency gains expressed in terms of the NNST to avert one death.

### 2.4. Number Needed to Self-Test

As pathogen ‘archetypes’, we selected Cholera, COVID-19, Mpox, Ebola and Influenza-A: all are directly transmitted infections that are priority pathogens for African Union preparedness efforts [1] and rank in the top 10 of the FIND Pathogen Diagnostics Readiness Index (PDxRI) [40], indicating technological plausibility of self-testing diagnostic intervention (Section S6).

For each pathogen, we assigned reference values for transmissibility (R_0_), severity (case fatality ratio), latent period, and duration of infectiousness, to estimate the NNST to avert one death. These archetypes are not intended as biologically faithful representations of specific pathogens, but rather as illustrative transmission-severity profiles used to explore how self-testing performance varies across contrasting epidemiological conditions.

The model was stratified into three tranches of diagnostic performance: high (specificity = 0.98, sensitivity = 0.99), medium (0.90, 0.99), and low (0.90, 0.95). Regimes approximate commonly cited optimal and acceptable operational ranges across Target Product Profiles for rapid diagnostic tests for epidemic-prone pathogens [26,41,42,43,44,45,46,47]. For each pathogen, we calculated the median NNST across all diagnostic performance and adherence regimes. We generated non-dominated Pareto plots of cumulative self-tests deployed versus deaths averted stratified by diagnostic performance regimes and post-test isolation adherence. Non-dominated solutions represent parameter combinations where no alternative scenario achieved greater mortality reduction with fewer self-tests. This approach allowed identification of the most resource-efficient intervention configurations across diagnostic performance regimes and post-test isolation adherence levels [48]. We decomposed cumulative self-test isolation events into true-positive and false-positive pathways and calculated the false-positive:true-positive isolation ratio among Pareto-optimal solutions.

### 2.5. Stakeholder Engagement

Pathogen-specific model outputs were reviewed with policymakers working on diagnostic access in Nigeria and at the World Health Organization. Consultations were used to contextualize interpretation of the model outputs, particularly the relevance of coverage–impact trade-offs for decision-making in resource-constrained African health systems to situate the analysis within current pandemic preparedness contexts. Stakeholders were identified pragmatically through existing professional and implementation networks linked to self-testing and diagnostic preparedness work (See acknowledgements).

## 3. Results

### 3.1. Global Sensitivity Analysis

The overall impact of self-testing on epidemic mitigation was modest. The median reduction in peak prevalence following the addition of self-testing was 1.9% (IQR: 0.4–5.8%) (Section S7).

**Figure 1:**
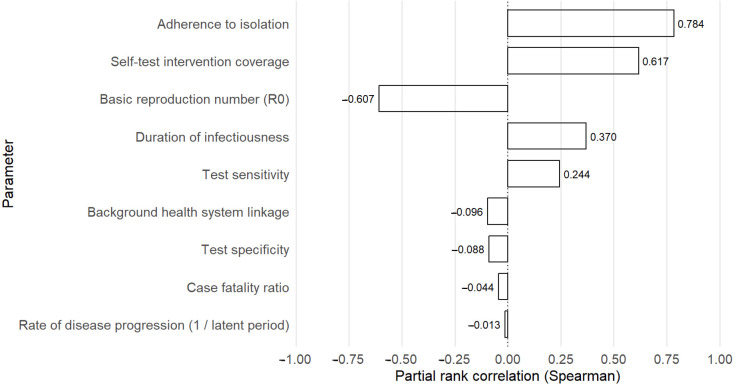
Partial Rank Correlation Coefficients for the relative difference in peak prevalence between SoC testing, and self-testing in addition to SoC, ranked by magnitude.

Global Partial Rank Correlation Coefficients indicated the strongest drivers of epidemiological impact were adherence to isolation measures (0.784) and the per capita rate of self-test =(0.617); that is, increasing adherence with isolation and increasing self-test intensity promoted the epidemiological impact of self-testing. As expected, lower values of $R_{0}$ (−0.607) were also highly correlated with impact. A longer infectious period (0.370) and greater test sensitivity (0.244) were also moderately correlated with impact of self-testing (Figure 1).

Stratifiying analyses across background rates of SoC testing (r) showed changes in the magnitude of correlations but no change in the overall direction of association or ranking (Section S5).

**Figure 2:**
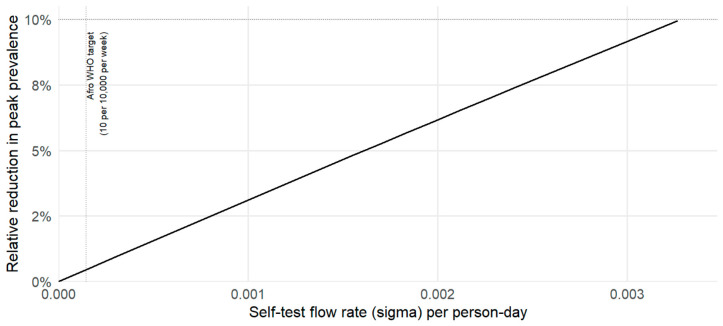
Rates of self-testing vs epidemiological impact at R_0_ = 1.1, under the lowest decile of background health-system capacity (r=0.0075), a favorable transmission and health-system scenario for self-testing impact. The WHO Afro COVID-19 target for all testing across health system (10 tests per 10,000 people per week) is shown with a vertical dotted line.

To explore thresholds for the rate of self-testing required for epidemic mitigation, we examined a favorable transmission and health-system scenario from which to evaluate the impact of self-testing: namely, a low value of $R_{0}$ (1.1) and a low value for background health-system testing capacity (0.0075). At the WHO Afro benchmark of 1.42 × 10^−4^ tests per person per day, the reduction in peak prevalence was only 0.45%. To achieve a 10% reduction, the required per capita self-testing rate was approximately 24 times higher than the WHO Afro benchmark, equivalent to around 0.0034 tests per person per day, or approximately 34 tests per 10,000 people per day. The reported threshold was estimated under median values for all remaining model parameters, including a test sensitivity of 78.7% and specificity of 99.3% (Figure 2).

Given isolation adherence was the strongest correlate of mitigation, we additionally examined the most optimistic upper-bound of 80% adherence to isolation. Even under this favorable adherence scenario, the rate of self-testing required to achieve a 10% reduction in peak prevalence remained above the WHO Afro benchmark, although appreciable reductions of 5% were obtainable by the WHO Afro benchmark (Section S3).

### 3.2. Marginal-Efficiency Gains

We next examined numbers needed to self-test (NNSTs) to avert one death across pathogen archetypes. These varied widely by pathogen type, from a median of 1512 tests per death for Ebola-like pathogens, to 355,708 tests per death for Mpox-like pathogens (Table 2).

Pareto frontiers showed approximately linear increases in deaths averted with increasing cumulative self-tests per person by epidemic end. However, absolute gains varied by pathogen. For Ebola-type pathogens, increasing cumulative testing intensity was associated with meaningful population-level mortality reductions, rising from approximately 7 to 8 deaths averted per 1000 people at three self-tests per person over the epidemic trajectory to over 20 deaths averted per 1000 people at nine tests per person over the epidemic trajectory. In contrast, for Mpox, Influenza A, and Cholera archetypes, mortality reductions remained small across the same range of testing intensity, remaining below ~0.2 deaths averted per 1000 people for Mpox and Influenza A, and below ~0.5 per 1000 people for cholera, even at the upper bound of testing explored. The Coronavirus archetype exhibited intermediate behavior, with cumulative testing up to approximately six tests per person associated with around 1–1.2 deaths averted per 1000 people.

**Figure 3:**
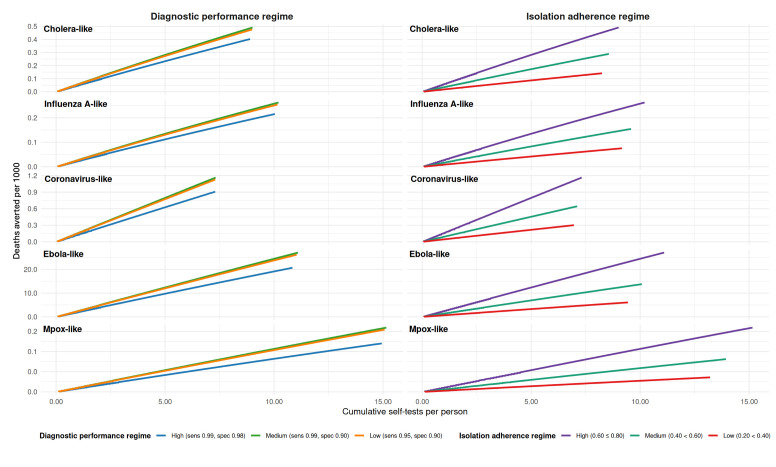
Non-dominated Pareto frontiers by diagnostic performance regime, adherence and pathogen.

Mortality gains were sensitive to diagnostic performance regime; across pathogens, the medium-performing diagnostic regime (sensitivity 0.99, and specificity at 0.90) yielded greater mortality reductions at comparable testing volumes than higher-specificity regimes (sensitivity 0.99, and specificity at 0.98). However, across pathogen archetypes, variation in post-test adherence with isolation produced substantially larger shifts in Pareto frontiers than variation in diagnostic performance (Figure 3).

False-positive: true-positive self-test isolation ratios differed between diagnostic regimes, with consistently higher false-positive ratios observed under the medium-performing regimes, although false positives outnumbered true positives in almost all scenarios (Table S3 in Sectin S7). Ratios were also pathogen-specific, ranging from high performance regime of 1:1 for Mpox-archetypes and Ebola-archetypes to 6.6:1 for Influenza A-archetypes, and from 4.8:1 for Mpox-archetypes and 5.2:1 for Ebola-archetypes to 33.1:1 for Influenza A-archetypes under the medium-performing regime.

Stakeholder-informed interpretation confirmed that different pathogen-archetypes would differ in the intensity of self-testing investment required, and that such differences (even at the scale and with the associated isolation burden presented here) would not preclude implementation at the national level, which was determined more by resource availability. However, stakeholders also queried population-level self-testing intervention given policy focus on targeted deployment of self-tests to priority populations to improve access where existing diagnostic systems are weakest (e.g., in the context of an epidemic, recent migrants to an area). Self-testing for epidemic response was also considered too nascent for explicit trade-off thresholds to be operationally meaningful. Self-testing was also not considered necessarily a full-epidemic trajectory diagnostic intervention, with potential benefit ascribed to its ability to be deployed rapidly in outbreaks compared to other more resource-intensive diagnostics. Stakeholders also noted that substantial additional investment (either through prior experience with self-testing in other disease areas or through community education alongside test distribution) would be required to support sustained uptake of diagnostics, and adherence with isolation measures across settings.

## 4. Discussion

The main findings from this modeling study were that untargeted population-wide self-testing linked to isolation is unlikely to function as a general epidemic-mitigation tool under conditions of constrained health-system capacity for the archetypes of the AU priority pathogens considered here (Ebola, Influenza A, Cholera, Coronavirus, and Mpox), as the testing volumes required to meaningfully mitigate epidemics exceed operationally plausible thresholds over a full-epidemic trajectory. When the outcome of interest shifts from mitigation to mortality reduction, however, self-testing showed incremental strategic value for high-severity pathogens with moderate transmission potential, such as Ebola archetypes, where small transmission reductions translate into meaningful survival gains. The value of self-testing was highly dependent on post-test behaviors; adherence to post-test isolation measures had greater influence on transmission reduction than test kit sensitivity or specificity changes.

That the epidemiological value of untargeted self-testing depends partially on pathogen characteristics is not surprising; interventions which shorten the effective infectious period are more impactful when outbreaks spread more slowly [49]. In our analysis, this was reflected by greater epidemic mitigation with lower R_0_ and longer duration of infectiousness. Pathogens with higher R_0_ and shorter duration of infectiousness spread more rapidly, outpacing diagnostic responses. In this context, successful self-testing strategies are most clearly established for pathogens such as HIV and Hepatitis C (HCV) that have infectious periods lasting many years and available effective treatment. By comparison, the fast-paced epidemic-prone pathogens considered in this study operate under very different dynamics. We show that the epidemic mitigation effects from untargeted self-testing will be minimal at the WHO Afro benchmark testing intensities (10 tests per 10,000 people per week). Our results concur with those from COVID-19 self-testing modeling studies, for example the Propelling Action for Testing and Treating (PATAT) framework [50] that estimated transmission impact in Zambia only if testing rates at least seven times higher than the WHO Afro benchmark intensity (defined above) were used under highly targeted distribution strategies.

However, our stakeholder analysis, reported here, also stresses the value that policymakers placed on the potential of self-testing to support rapid and early decentralized diagnostic access during outbreaks even if initial intensity was not operationally sustainable over the full epidemic trajectory. This can be best illustrated with the Ebola archetype. Although the level of self-testing coverage required to disrupt transmission chains exceeded the coverage threshold anchored to COVID-19 testing realities, coverage intensities above the WHO Afro benchmark intensities may be operationally plausible for initial outbreak management strategies. Given the clustered spatial dynamics of Ebola outbreaks, it is theoretically possible that small transmission networks could be saturated with self-tests, prior to the establishment of molecular testing and formal isolation infrastructure in naïve communities [51,52]. A target distribution model for this intervention could be through using the formal and informal community systems within villages that are close to, but not yet affected by, the outbreak [53,54], or through deployment after funerals as an enhanced form of contact tracing given the substantial transmission burden attributed to unsafe burial practices during previous outbreaks [55,56,57]. Absolute mortality gains were also observed at lower testing numbers for higher-severity pathogen archetypes, mediated through transmission effects, meaning rapid deployment of these decentralized diagnostics also confers absolute mortality benefit.

Given that Ebola is a highly stigmatized disease, which delays presentation for care seeking [58,59], the provision of a home-based distribution strategy prior to outbreak in neighbouring villages may be a socially acceptable intervention. An oral rapid diagnostic kit exists [60], made by the same manufacturer using the same oral-saliva testing modality that was successfully deployed in the HIV response [4]. This remains highly speculative—and would require rethinking broader post-test response protocols, including quarantine arrangements for people who test positive at home, access to safe confirmatory tests for individuals and the biosafety of specimen handling at the point of test conduct—but it represents an interesting area for future research.

Conversely, the high thresholds for incremental mortality gains for several pathogen archetypes (Cholera, Mpox and Influenza-A) were not interpreted by policymakers as rendering these unsuitable for self-testing deployment. Rather, discussions emphasized the relative weighting placed on access amongst underserved populations in the context of under-resourced health systems rather than absolute mortality gains. Cost-utility decisions will require a far more systematic stakeholder consultation, including many of the global donors who fund and support self-testing regulation [61,62]. Nonetheless, if frameworks do not explicitly tie diagnostic investment to impact through access, the question of rational use of self-test kits for outbreak response remains, especially in the context of under-resourced health systems.

Finally, the analysis found that post-test isolation adherence outstripped the relative importance of test accuracy: in the global sensitivity analysis, adherence was the dominant correlate with epidemic mitigation, and in the Pareto optimization for deaths averted, variation in isolation adherence produced substantially larger shifts in the potential mortality gains for increasing the number of self-tests than variation in diagnostic performance. Post-test isolation adherence was further emphasized by policymakers consulted in this analysis as a fundamental consideration for deployment of any self-testing intervention, shaped by prior exposure to testing and community understanding of post-test expectations, rather than by test availability alone.

This matters because if the search for the perfect self-test from an epidemiological perspective is less important than an individual’s ability to adhere—or otherwise—with the self-test result, then perhaps considerations of post-test isolation adherence should be included in diagnostic preparedness frameworks. This is not to downgrade the importance of test accuracy; social science enquiry on COVID-19 self-testing in Nigeria highlighted that considerations of post-test isolation adherence were not necessarily divorced from technical considerations of test accuracy, with policymakers, implementers, and healthcare workers highlighting that public-level belief in test accuracy was a crucial enabler of isolation adherence [63]. We highlight the potential for a substantial false-positive burden. Although stakeholders did not frame a trade-off in terms of isolation burden, the widespread experience of lockdown fatigue across the African continent during the COVID-19 pandemic suggests that repeated isolation of otherwise healthy individuals may reduce the acceptability of self-testing interventions and undermine adherence over time [64]. However, the acceptability of a false-positive isolation is also likely pathogen-specific. For example, during the 2014–2016 Ebola outbreak in Guinea, relatively non-specific community quarantine measures were considered more acceptable when individuals perceived Ebola as highly lethal, believed themselves to be at risk of infection, and had access to basic support during quarantine [65].

Nevertheless, there are clear ethical reasons for stringency on test accuracy especially given the findings here with the potential of self-testing for Ebola-type pathogens. With Ebola, there is potential for substantial and significant harm associated with false-positive results, as these require quarantine with other infected individuals, that render test specificity paramount [43]. However, insomuch as values and preferences for test type, and lay usability studies, have all been included as components of self-testing diagnostic access and target product profiles [14,15], what this analysis surfaces is the potential need to incorporate explicit considerations of post-test isolation adherence within diagnostic preparedness planning, especially given stakeholder interpretation that there are mechanisms available from public health programming to support individuals’ capacity to isolate.

### Limitations

This analysis aimed to identify generalizable conditions under which self-testing may have epidemiological value given the current limited inclusion of self-testing in diagnostic preparedness frameworks. However, in so doing, this approach has necessary limitations; pathogen archetypes are not intended to represent specific diseases, and results should not be interpreted as empirical effectiveness estimates for any given pathogen. We use simplified pathogen archetypes, test kit performance, and behavioral response to establish general principles with this conceptual model.

We recognize, for example, that non-nucleic specific antigen-detection tests (Ag-RDT), which identify pathogen antigens, have emerged as promising diagnostics for decentralization and potentially self-testing [22,41,44,66,67,68]. With Ag-RDTs, sensitivity varies over time, corresponding with the viral load trajectory [31] and hence with peak infectiousness, as was especially well documented for SARS-CoV-2 [69]. Similar arguments for important time-varying properties can be made for adherence to isolation. For pathogens requiring prolonged isolation, or where false-positive results generate substantial unnecessary isolation, adherence may decline over time. Conversely, adherence may increase during periods of greater prevalence or mortality. Modeling these processes would require pathogen-specific behavioral assumptions therefore representing an important future extension into pathogen-specific modeling.

Another important limitation is that we do not consider the benefits of treatment for either individual health or for transmission interruption: in our model, isolation is the sole mechanism through which self-testing generates benefit. Clearly, this underestimates the mortality benefits of self-testing impact for conditions such as Ebola and Cholera, where treatments can reduce the case fatality ratio (CFR). Future modeling of treatment-mediated pathways would enable pathogen specific assessments of the mortality benefits of self-testing.

We also do not consider targeted self-testing strategies within heterogeneous transmission networks. The current model assumes homogeneous mixing and population-wide deployment; it does not consider targeted self-test distribution strategies, such as contact tracing and social network distribution within risk groups. An explicit network or metapopulation model may identify more efficient deployment strategies and consequently lower NNST estimates than those reported under population-wide deployment assumptions for clustered transmission networks such as Ebola or Mpox.

Finally, we have not considered epidemic surveillance needs, as these are beyond the scope of this analysis, but recognize their importance especially where genomic or variant-level information is required. In practice, surveillance using centralized and decentralized laboratory platforms will need to be factored into confirmatory testing pathways [70].

Despite these limitations, our results highlight the need to carefully tailor self-testing interventions to pathogen attributes, and the critical role of isolation adherence.

## 5. Conclusions

The main findings from this modeling study suggest that self-testing is unlikely to function as a general epidemic-mitigation tool under conditions of constrained health-system capacity, as the testing volumes required to meaningfully reduce transmission exceed operationally plausible thresholds over a full epidemic trajectory. However, future network modeling or demonstration studies evaluating rapid, early decentralized self-testing deployment during Ebola-archetype outbreaks may identify operationally feasible coverage intensities capable of supporting epidemic mitigation and reducing mortality, warranting further investigation.

## Figures and Tables

**Table 1:** Externally specified parameters, plausible ranges and rationale.

Parameter	Plausible Ranges	Rationale
Disease-specific parameters		
Basic reproduction number, R_0_	1.1 to 10	Minimum threshold for epidemic propagation (R_0_ > 1) up to highly transmissible airborne infections (R_0_ = 10).
Case Fatality Rate (CFR)	1% to 50%	Moderate-severity respiratory infections (e.g., seasonal influenza [26]) to high-severity viral hemorrhagic fevers (e.g., Ebola [27]).
Latent period (1/ε)	1 to 12 days	Considering acute outbreak-potential pathogens only, ranging from short-generation infections (e.g., Influenza A [28]) to longer latent infections (e.g., Mpox [29,30]).
Duration of infectiousness (D)	3 to 21 days	As above.
Device-specific parameters		
Sensitivity and specificity of self-testing	Draws from plausible bivariate distribution; specificity capped above at 0.90	Empirically derived sensitivity–specificity sampling draws from a bivariate random effects model for self-test kits with available accuracy data (*N* = 91 [31,32,33,34,35]). Specificity capped above at 0.90 to avoid rewarding devices with sub-optimal specificity (peak prevalence will decline with increased false-positive isolation).
Behavioral parameters		
Post-test adherence with isolation measures (a)	0 to 0.8	Systematic review evidence suggests adherence with post-test isolation clusters well below full adherence [36]. Adherence ranges from 0 up to 0.8 (upper plausible bound for sustained isolation adherence in outbreak settings).
Intervention coverage parameters		
Per capita rate of self-testing (σ)	0 to 0.15 per person per day	No testing to the maximum global daily testing conducted during the COVID-19 pandemic (Cyprus on 9 January 2022 at 0.15 per person per day [37]). During the COVID-19 pandemic an overall per capita testing rate of 1.42 × 10^−4^ per person-day (10 tests per 10,000 people per week) was identified as the minimum overall testing threshold required to disrupt transmission chains by WHO Afro. Only 16 countries achieved this benchmark by mid-2021 [20].
Health-system context		
Rate at which health system links people into isolation (r)	0 to 0.15	Rate depends on duration of infectiousness (D), as: $P=1-e^{-rD}$ where *P* is the overall probability of linkage to isolation during the disease course, r is the daily hazard of detection and linkage, and *D* is the duration of infectiousness [38]. At maximum rate (0.15) and maximum duration of infectiousness (21 days), the health system would link 96% people into isolation. At a rate of *r* = 0.007 corresponding to a minimum duration of infectiousness of 3 days, it would link 2% of infections, a detection rate not unheard of during the early stages of the COVID-19 pandemic [39].

**Table 2:** Median (interquartile range) number of self-tests required to avert one death, ranked by median, for a self-testing intervention in addition to standard-of-care testing under the lowest decile background health-system case-detection rate (r=0.0075); estimates are pooled across diagnostic performance regimes, adherence estimates (0.2–0.8) and self-test flowrates up to 0.015 per person-day.

Pathogen Archetypes	Median Number Needed to Self-Test (NNST) to Avert One Death	IQR (25%)	IQR (75%)
Ebola-like	1512	726	5430
Coronavirus-like	22,590	11,001	74,619
Cholera-like	55,453	28,920	182,469
Influenza A-like	117,231	60,529	406,948
Mpox-like	355,708	171,447	1,268,315

Median estimates exclude *N* = 845 of 65,190 simulations. These outputs were omitted because they showed either no deaths averted (*N* = 615) or negative deaths averted (*N* = 230). Outlier checks indicated numerical artefacts, with <1 × 10^−9^ difference between baseline and intervention outcomes and identical epidemic-end days.

## Data Availability

The full model code is available at https://github.com/yasmindunkley/self-test-epidemic/releases/tag/v1.0, accessed on 3 March 2026, archived at 10.5281/zenodo.18846426.

## Supplementary Materials

The following supporting information can be downloaded at: https://www.mdpi.com/article/10.3390/diagnostics16132092/s1, Section S1: Supplementary materials; Section S2: Diagnostic accuracy draws; Section S3: Stratification by health system capacity; Section S4: Pathogen archetype modelling logic; Section S5: Empirical cumulative distribution function of relative difference in peak prevalence; Section S6: Self-test flow rates to achieve epidemic mitigation at R_0_ = 1.1, under the lowest decile of background health system capacity (*r* = 0.0075) stratified by optimum adherence to isolation (0.8); Section S7: False-positive and true-positive isolation burden across Pareto-optimal diagnostic regimes. References [35,71] are cited in the Supplementary Materials.

## Abbreviations

The following abbreviations are used in this manuscript:

AU	African Union
CFR	Case Fatality Rate
COVID-19	Coronavirus Disease 2019
HCV	Hepatitis C Virus
HIV	Human Immunodeficiency Virus
IQR	Interquartile Range
LHS	Latin Hypercube Sampling
NNST	Number Needed to Self-Test
PATAT	Propelling Action for Testing and Treating
PDxRI	Pathogen Diagnostics Readiness Index
PRCC	Partial Rank Correlation Coefficient
SEIR	Susceptible–Exposed–Infectious–Recovered
SoC	Standard of Care
SROC	Summary Receiver Operating Characteristic
ST	Self-Testing
WHO	World Health Organization
WHO Afro	World Health Organization Regional Office for Africa
