# Supplementary material for: Epidemic Mitigation and Marginal Mortality Gains Using Self-Testing as a Diagnostic Intervention for Epidemic-Prone Diseases in Africa
Source: Diagnostics (Basel). 2026 Jul 3;16(13):2092. doi: 10.3390/diagnostics16132092 (PMC13360251; doi:10.3390/diagnostics16132092)
Supplement: Supplementary file 1 [file diagnostics-16-02092-s001.zip › diagnostics-4352664-supplementary.pdf]

diagnostics MDPI

S1. Supplementary materials

S1.1. Model specification

Figure 1. Conceptual model of a self-testing intervention on cases averted and deaths averted

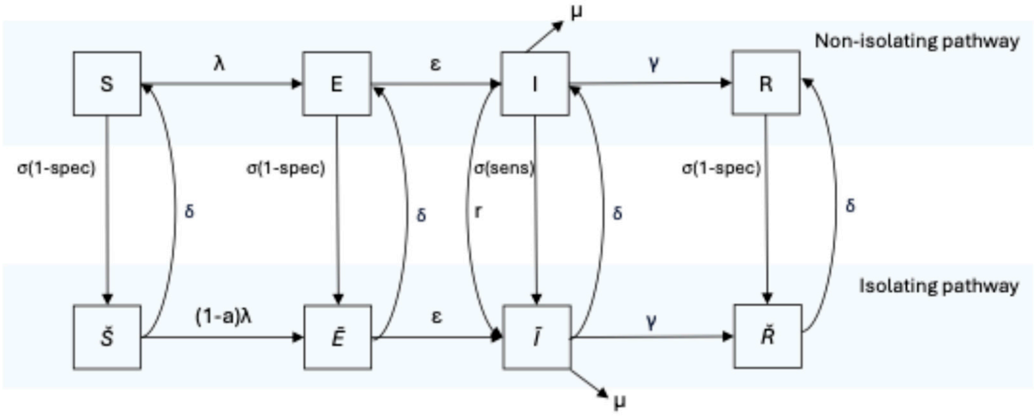

Figure S1. Conceptual model governing the impact of self-testing as an addition to standard of care (SoC) testing provision.

S1.2. Governing equations and full model specification

Table S1. Parameter derivations

Parameter	Name	Derivation equation
Population specific parameters		
N	Population in the model	S + E + I + R + Si + Ei + Ii + Ri
Disease specific parameters		
β	Transmission rate	R0(γ + μ)
β_i*	Transmission in isolation	β(1 − a)
λ	Force of infection (non-isolated)	$\frac{\beta I + \beta_i I_i}{N}$
$\tilde{\lambda}$	Force of infection (isolated)	(1−a)λ
μ	Mortality rate	$\frac{\mathrm{CFR}}{D}$
γ	Recovery rate	$\mu\frac{(1-\mathrm{CFR})}{CFR}$
Intervention coverage parameters		
d	Duration of isolation	D × 1.5 (1.5 times the duration of infectiousness)
δ*	Release rate from isolation	$\frac{1}{(\mathrm{a}\cdot\mathrm{d})}$

*Adherence (a) influences the model through three pathways. First, it reduces transmission from infectious individuals in isolation through the term $\beta_i = \beta(1-a)$. Second, it reduces the force of infection experienced by susceptible individuals in isolation through $\tilde{\lambda} = (1-a)\lambda$. Third, it determines the duration of isolation through the release rate $\delta = 1/(a \cdot d)$, such that lower adherence results in earlier exit from isolation. Thus, adherence jointly influences both the effectiveness and duration of isolation.

S1.3. *State variable differential equations*

Non isolating pathway

$$\frac{dS}{dt} = \delta Si - \lambda S - \sigma(1 - spec)S$$

$$\frac{dE}{dt} = \lambda S - \sigma(1 - spec)E - \varepsilon E + \delta Ei$$

$$\frac{dI}{dt} = \varepsilon E - \sigma(sens)I - \mu I - \gamma I - rI + \delta Ii$$

$$\frac{dR}{dt} = \gamma I - \sigma(1 - spec)R + \delta Ri$$

Isolating pathway

$$\frac{dSi}{dt} = \sigma(1 - spec)S - \delta Si - (1 - a)\lambda Si$$

$$\frac{dEi}{dt} = \sigma(1 - spec)E - \varepsilon Ei + (1 - a)\lambda Si - \delta Ei$$

$$\frac{dIi}{dt} = \varepsilon Ei + \sigma(sens)I - \mu Ii - \gamma Ii - \delta Ii + rI$$

$$\frac{dRi}{dt} = \gamma Ii + \sigma(1 - spec)R - \delta Ri$$

Output specification

Cumulative Mortality

$$\frac{dCM}{dt} = \mu I + \mu Ii$$

Cumulative isolation events

$$\frac{dCIso}{dt} = \underbrace{\sigma(1 - spec)(S + E + R)}_{\text{false-positive self-test isolation}} + \underbrace{\sigma(sens)I}_{\text{true-positive self-test isolation}} + \underbrace{rI}_{\text{SoC isolation}}$$

Cumulative false positive selftesting events

$$\frac{dCIsoFP}{dt} = \sigma(1 - spec)(S + E + R)$$

Cumulative true positive selftesting events

$$\frac{dCIsoTP}{dt} = \sigma(sens)I$$

Initial conditions were:

$S(0) = 1 - 10^{-6}$, $I(0) = 10^{-6}$, and $E(0) = R(0) = S_i(0) = E_i(0) = I_i(0) = R_i(0) = CI(0) = CM(0) = CIso(0) = CIsoFP(0) = CIsoTP(0) = 0$

S1.4. *Outcome specification*

1. Peak prevalence reduction

The reduction in epidemic peak infectious prevalence between the intervention and baseline (standard-of-care) scenarios was calculated as:

$$\Delta_{\text{peak,abs}} = \max(I_T^{(0)}(t)) - \max(I_T^{(1)}(t))$$

$$\Delta_{\text{peak,rel}} = 1 - \frac{\max(I_T^{(1)}(t))}{\max(I_T^{(0)}(t))},$$

- where $I_T(t) = I(t) + I_i(t)$represents total infectious prevalence at time t.
- where $0, 1$ represent baseline (0) and intervention runs (1) respectively.

Outbreak mitigation was defined operationally as a reduction in peak prevalence, that is, $\Delta_{\text{peak,rel}} > 0$

2. Number Needed to Self-Test (NNST)

The NNST quantified the relative resource intensity of self-testing required to avert additional deaths:

$$NNST = \frac{Tests_{\text{total}}^{(1)}}{CM^{(0)}(t^{*(0)}) - CM^{(1)}(t^{*(1)})}$$

where:

- $Tests_{\text{total}}^{(1)}$is the total number of self-tests conducted during the intervention, calculated daily as
$$\sigma[\text{S(t)} + \text{E(t)} + \text{I(t)} + \text{R(t)}]$$
and summed to the intervention hard-stop or maximum duration as per the model logic.
- $CM^{(k)}(t^{*(k)})$ is the cumulative mortality at the hard-stop time or maximum duration $t^{*(k)}$ in baseline and intervention respectively.

3. Supplementary assessment of false-positive isolation burden

To contextualise the diagnostic specificity trade-offs observed in the Pareto optimisation, cumulative self-test isolation events were decomposed into false-positive and true-positive isolation events. The false-positive:true-positive (FP:TP) self-test isolation ratio was calculated as:

$$FP{:}TP = \frac{CIso_{FP}^{(1)}(t^{*(1)})}{CIso_{TP}^{(1)}(t^{*(1)})}$$

where:

- $CIso_{FP}^{(1)}(t^{*(1)})$is the cumulative number of false-positive self-test isolation events at the intervention hard-stop time or maximum duration.
- $CIso_{TP}^{(1)}(t^{*(1)})$is the cumulative number of true-positive self-test isolation events at the intervention hard-stop time or maximum duration.

This ratio represents the number of false-positive self-test isolation events occurring for every true-positive self-test isolation event. Ratios were calculated for Pareto-optimal solutions.

S1.5. *Modelling assumptions and time horizons*

The model assumes a closed, homogeneous population scaled to N = 1. Births, non-disease mortality, and demographic turnover are not modelled, as the simulated outbreak duration is short relative to demographic timescales (maximum duration of 1,825 days). Simulations were seeded with one infectious case, corresponding to an initial prevalence of 10^{-6} in the I compartment, with all other state variables initialised at zero. Simulations advanced in daily time steps until either sustained epidemic fade-out or 1,825 days (approximately five years).

Epidemic fade-out was reached once a daily incidence of below 10^{-6} was obtained for 100 consecutive days, mirroring the initial seeding of infection at 10^{-6}. A 7-day moving average was applied to daily incidence to smooth short-term fluctuations (k-point smoothing) and enable consistent identification of epidemic fade-out. Testing was maintained throughout this 100-day period. The 100-day period was chosen to reflect the 100-days mission; operational guidance that pandemic countermeasures should be ready for deployment within roughly 100 days [1] to prevent rebound once the testing intervention was stopped e.g., novel therapeutics/ vaccination. A minimum simulation horizon, set at ten combined latent-plus-infectious periods, ensured that slow-developing epidemics were not misclassified as successful suppression before they were established.

The model was coded in R and independently implemented in Python to verify consistency and reproducibility across simulations.

S2. Diagnostic accuracy draws

A plausible sampling region in the sensitivity–specificity space was defined using a bivariate random-effects (Reitsma [2]) model fitted to diagnostic accuracy data for HIV and COVID-19 self-testing devices. All available self-test accuracy studies were pooled irrespective of disease, with the objective of identifying an empirically grounded sensitivity–specificity region rather than deriving disease-specific accuracy estimates (N = 94 accuracy results [3-7]) (**Figure S2**).

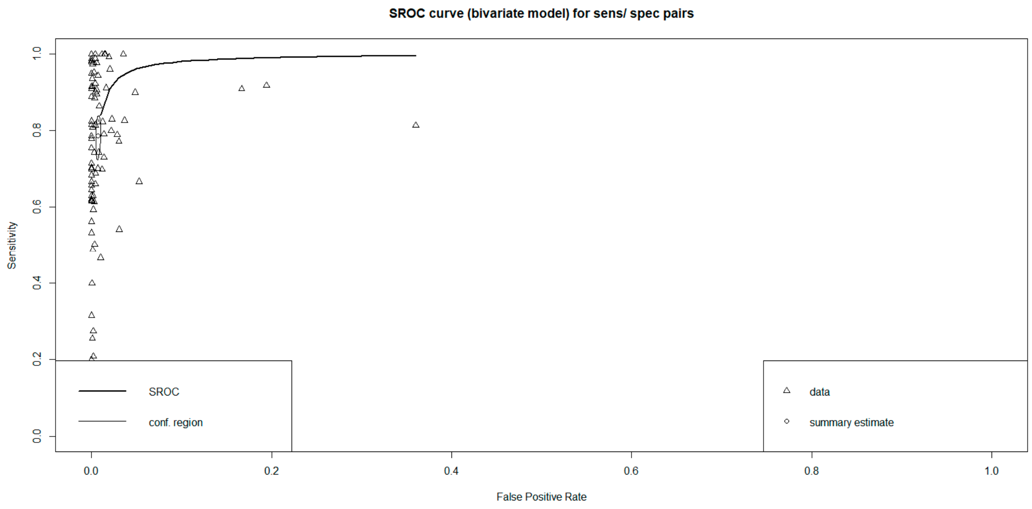

Figure S2. SROC curve from the Reitsma bivariate random-effects model using combined HIV and COVID-19 self-test accuracy data (N = 94 [3-7]). The curve and confidence region describe the empirically supported sensitivity–specificity space used to guide sampling.

We fitted the Reitsma model to the full dataset and extracted the between-study mean vector and variance–covariance matrix for logit-sensitivity and logit-false positive rate. We then generated an over-sampled set of joint draws from this bivariate normal

random-effects distribution, transformed them back to the probability scale using the inverse logit, and applied rejection sampling to retain only draws with specificity ≥0.90, consistent with minimum performance expectations for current self-tests. Sampling proceeded until 20,000 accepted (sensitivity, specificity) pairs were obtained. These correlated accuracy pairs were assigned directly to the Latin hypercube samples in the transmission model, preserving the empirical dependency structure while constraining the sampling region to high-specificity tests.

S3. Stratification by health system capacity

Stratifying analyses across deciles of background health-system case-detection capacity showed that the magnitude of PRCCs attenuated as background linkage increased, consistent with reduced marginal benefit of self-testing when standard-of-care linkage is stronger. However, the direction and relative ranking of the principal correlates of peak-prevalence reduction remained unchanged across deciles. Adherence to isolation, self-test intervention coverage, R_0, duration of infectiousness, and test sensitivity remained the dominant drivers. This suggests that the model conclusions are robust across very low to high background linkage settings, including scenarios approximating minimal routine health-system detection.

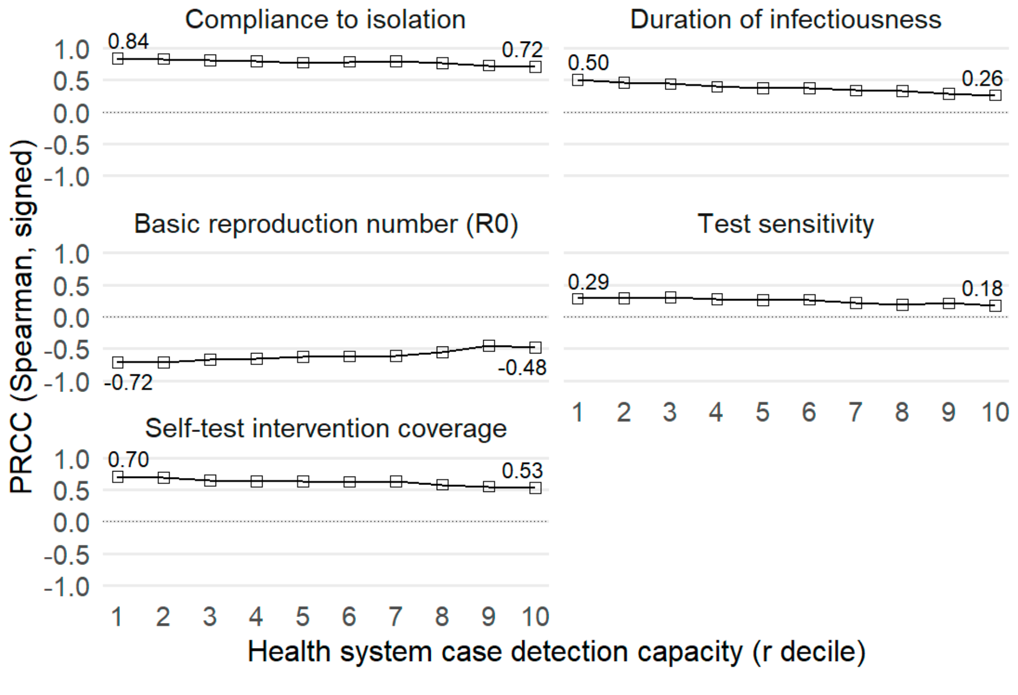

Figure S3. Stratified analysis of robustness of Partial Rank Correlation Coefficients of existing correlates of reductions in peak prevalence against increasing health system case detection capacity.

S4. Pathogen archetype modelling logic

Given the skewed nature of biological parameters, medians were preferred instead of means. Where multiple estimates were available, we selected a value within the published range using a hierarchy of evidence to ensure parameter consistency across pathogens (privileging systematic reviews and meta-analyses, followed by current WHO or Lancet disease profiles/factsheets, and subsequently aggregated real-world data from past epidemics used in established models when higher-level evidence was not available). The analysis was not intended to reproduce specific disease dynamics but evaluate how efficiency varies across transmissibility–severity archetypes (**Table S2**).

Table S2. Pathogen archetypes, ranked by Pathogen Diagnostics Readiness Index (PDxRI).

Pathogen-archetype	Transmission-modality	PDxRI [31]	R_0	Duration of infectiousness	CFR
Coronavirus	Respiratory droplets and aerosol	100	2.4 [32]	10 days [33]	3.6% [34]
Influenza A†	Respiratory droplets and aerosols	56.58	1.3 [17]	5 days [17,35]	0.5% [19]
Mpox (Orthopoxvirus	Physical contact, body fluids, respiratory droplets, sexual	47.11	2.5 [20]	25 days [21]	0.19% [20][ii]
Cholera	Faecal-oral	31.26	1.35 [36]*	5 days [37]	1% [37]
Ebola virus disease (Zairense and sudanese)	Bodily fluids	21.44	1.95 [38]	15 days [39][i]	60% [18]

PDxRI FIND Pathogen Diagnostics Readiness Index; indicates the level of existing availability of diagnostic tools that renders self-testing diagnostic intervention technologically plausible.
†Pandemic influenza has only been caused by influenza A sub-types (viruses H1, H2, H3). These viruses originated from avian or swine reservoirs but are defined by sustained human-to-human transmission via respiratory droplets and aerosols. Pooled Influenza A estimates are used here.
* Informed through past epidemics (i.e., a mean of Zimbabwe & Haiti; Zimbabwe:1.15 & Haiti: 1.55.) However, when calculated at a provincial/departmental level, estimated basic reproductive numbers were highly heterogeneous, with a range of 1.11 to 2.72 in Zimbabwe and 1.06 to 2.63 in Haiti [36].
i Unlike other pathogens with published infectious periods, we use onset to discharge from the WHO data as a proxy for duration of infectiousness [39].
ii Estimated from the 2022-23 epidemic [20].

Marginal-efficiency analyses were conducted within the WHO African Region minimum testing benchmark reported during the COVID-19 pandemic (10 tests per 10,000 people per week; $\sigma \approx 1.42 \times 10^{-4}$ per person-day). Self-testing flow rate (σ) was varied from zero up to 0.0148 per person-day, corresponding to approximately two orders of magnitude above this benchmark. This envelope was used to characterize how deaths averted behave under scaled increases in testing capacity and does not represent operationally feasible deployment levels. Adherence was varied from 0 to 0.8 in 0.02 increments.

S5. Empirical cumulative distribution function of relative difference in peak prevalence

The distribution of relative differences in peak prevalence (intervention vs baseline) was highly right-skewed, indicating clustering around 0, with a small number of simulations resulting in larger reductions (**Figure S4**).

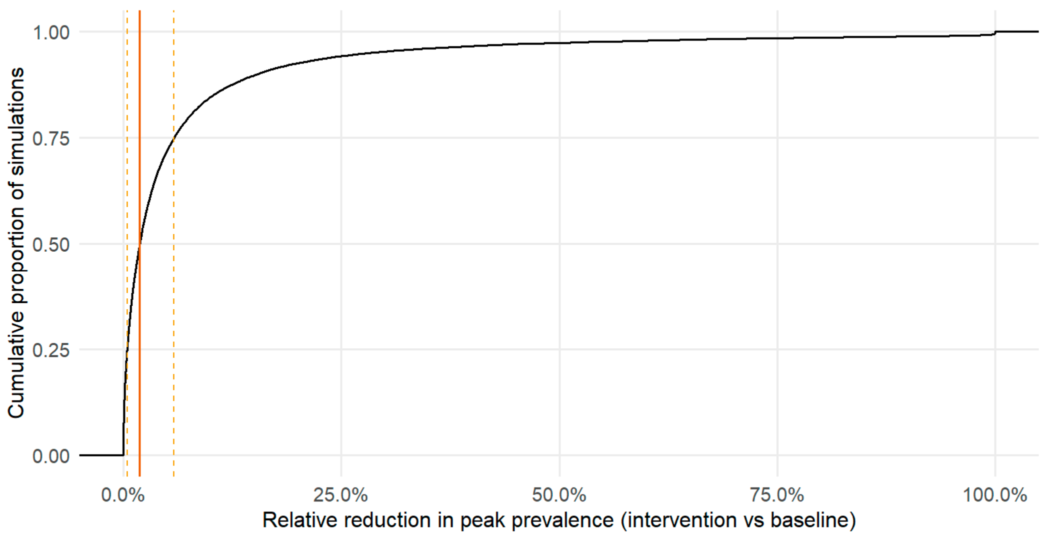

Figure S4. Empirical cumulative distribution function of relative difference in peak prevalence across simulations. Solid orange line represents median value, with dotted line interquartile range.

S6. Self-test flow rates to achieve epidemic mitigation at R0 = 1.1, under the lowest decile of background health system capacity ($r = 0.0075$) stratified by optimum adherence to isolation (0.8)

Even under a favorable compliance scenario, the self-test flow rate required to achieve a 10% reduction in peak prevalence remained above the WHO African Region benchmark, although appreciable reductions of 5% were obtainable by the WHO African Region benchmark (Figure S5)

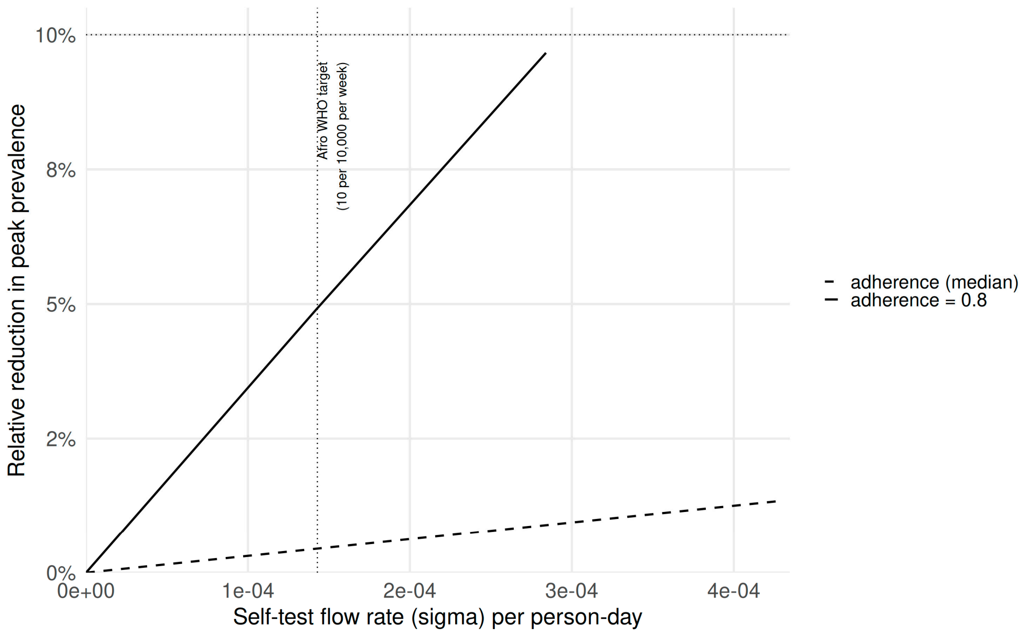

Figure S5. Self-test flow rates to achieve epidemic mitigation at R0 = 1.1, under the lowest decile of background health system capacity ($r = 0.0075$) stratified by optimum adherence to isolation (0.8). The WHO African Region COVID-19 target for all testing across health system (10 tests per 10,000 people per week) is included with a dotted line.

S7. False-positive and true-positive isolation burden across Pareto-optimal diagnostic regimes

To contextualise the diagnostic specificity trade-offs observed in Figure 3, cumulative self-test isolation events were decomposed into true-positive self-test isolations, and false-positive self-test isolations. False-positive:true-positive self-test isolation ratios were then calculated for Pareto-optimal solutions within the high- and medium-diagnostic performance regimes (**Table S3**)

Table S3. False-positive:true-positive self-test isolation ratios amongst Pareto-optimal solutions for high- and medium-diagnostic performance regimes.

Pathogen	High diagnostic performance regime (FP:TP)	Medium diagnostic performance regime (FP:TP)
Cholera	5.2:1 (5.1–5.7)	26.5:1 (25.5–29.1)
Coronavirus	1.2:1 (1.1–1.2)	5.9:1 (5.7–6.3)
Ebola	1.0:1 (1.0–1.1)	5.2:1 (4.9–5.9)
Influenza A	6.6:1 (6.3–7.2)	33.1:1 (31.7–36.9)
Mpox	1.0:1 (0.9–1.1)	4.8:1 (4.5–5.7)

FP:TP ratios were consistently higher under the medium diagnostic performance regime (sensitivity 0.99, specificity 0.90) than under the high diagnostic performance regime (sensitivity 0.99, specificity 0.98) across all pathogen archetypes.

References

1. International Pandemic Preparedness Secretariat. 100 Days Mission. Available online: https://ippsecretariat.org/ (accessed on 31st October 2025).
2. Reitsma, J.B.; Glas, A.S.; Rutjes, A.W.; Scholten, R.J.; Bossuyt, P.M.; Zwinderman, A.H. Bivariate analysis of sensitivity and specificity produces informative summary measures in diagnostic reviews. *J Clin Epidemiol* **2005**, *58*, 982–990, doi:10.1016/j.jclinepi.2005.02.022.
3. Brümmer, L.E.; Katzenschlager, S.; McGrath, S.; Schmitz, S.; Gaeddert, M.; Erdmann, C.; Bota, M.; Grilli, M.; Larmann, J.; Weigand, M.A.; et al. Accuracy of rapid point-of-care antigen-based diagnostics for SARS-CoV-2: An updated systematic review and meta-analysis with meta-regression analyzing influencing factors. *PLoS Med* **2022**, *19*, e1004011, doi:10.1371/journal.pmed.1004011.
4. Wei, C.; Yan, L.; Li, J.; Su, X.; Lippman, S.; Yan, H. Which user errors matter during HIV self-testing? A qualitative participant observation study of men who have sex with men (MSM) in China. *BMC Public Health* **2018**, *18*, 1108, doi:10.1186/s12889-018-6007-3.
5. Tonen-Wolyec, S.; Sarassoro, A.; Muwonga Masidi, J.; Twite Banza, E.; Nsiku Dikumbwa, G.; Maseke Matondo, D.M.; Kilundu, A.; Kamanga Lukusa, L.; Batina-Agasa, S.; Bélec, L. Field evaluation of capillary blood and oral-fluid HIV self-tests in the Democratic Republic of the Congo. *PLoS One* **2020**, *15*, e0239607, doi:10.1371/journal.pone.0239607.
6. Kumwenda, M.; Indravudh, P.; Johnson, C.; Sinjani, G.; Choko, A.; Nundwe, S.; Mee, P.; Nzawa, R.; Nyirenda, R.; Kandulu, J.; et al. Uptake and performance of blood-based self-testing versus oral fluid-based self-testing in Blantyre district, Malawi. In Proceedings of the 10th IAS Conference on HIV Science (IAS 2019), Mexico City, Mexico, 2019/07/21, 2019.
7. World Health Organization. Guidelines on HIV self-testing and partner notification: supplement to consolidated guidelines on HIV testing services. Available online: https://apps.who.int/iris/handle/10665/251655 (accessed on
